# Spinal Deformity Manifested by Urinary Tract Infection: A Case of Acute-on-Chronic Mobility Dysfunction

**DOI:** 10.7759/cureus.55475

**Published:** 2024-03-04

**Authors:** Noriko Yamasaki, Junki Mizumoto, Taro Shimizu

**Affiliations:** 1 Center for Medical Training, Ehime Seikyo Hospital, Matsuyama, JPN; 2 Department of Medical Education Studies, International Research Center for Medical Education, Graduate School of Medicine, The University of Tokyo, Tokyo, JPN; 3 Department of Diagnostic and Generalist Medicine, Dokkyo Medical University Hospital, Mibu, JPN

**Keywords:** diagnostic excellence, neurogenic bladder dysfunction, horizontal tracing, vertical tracing, urinary tract infection, diabetes mellitus, diffuse idiopathic skeletal hyperostosis, ossification of the posterior longitudinal ligament

## Abstract

A 53-year-old man with diabetes mellitus presented to the emergency department with a fever and impaired mobility. A preliminary diagnosis of urinary tract infection was made based on dysuria and pyuria. History-taking revealed a history of gait disturbance and difficult urination. A thorough physical examination suggested a spinal abnormality. MRI scan revealed a narrow spinal canal due to ossification of the posterior longitudinal ligament and diffuse idiopathic skeletal hyperostosis. Throughout the diagnostic process, we employed both vertical tracing to investigate the causes of urinary tract infection and horizontal tracing to explore comorbidities such as diabetes. Additionally, we introduced appropriate social security and support systems under the name of diagnostic excellence.

## Introduction

Immobility frequently accompanies fever in patients [1]. Nonetheless, thorough history-taking, physical examination, and diagnostic strategies enable physicians to identify underlying issues. Here, we present a case of a febrile patient confined to bed at home due to multifactorial reasons and diagnosed as a urinary tract infection. The patient's history of diabetes mellitus, gait disturbance, urinary incontinence, and neurological abnormalities suggested myelopathy originating from spinal regions. It is crucial for physicians to recognize that acute events can exacerbate pre-existing mobility limitations, leading to a scenario of acute-on-chronic mobility dysfunction.

## Case presentation

A 53-year-old man presented to the emergency department with a fever and impaired mobility. He was well until four days prior to the presentation when his fever developed without any reason coming to his mind. He reported that his mentation was intact, albeit he felt malaise. The patient denied any symptoms, including sore throat, cough, dyspnea, chest pain, abdominal pain, back pain, extremity pain, or numbness. He attempted to seek medical attention but fell out of bed and remained incapacitated on the floor because of fatigue and weakness. He lived alone and immobilized at that time, so he could not call for help. Thankfully, three days later, the landlord visited him by chance and discovered him lying on the floor, soaked in urine and feces. The patient was transferred to our hospital for evaluation and management.

The patient reported difficulty in ascending stairs, frequent tripping, and recurrent fall over several years. He also complained of bilateral plantar numbness. Additionally, he had been dealing with urinary incontinence at night for the last six months. His urination was associated with small volumes, weak intensity, and a sensation of residual urine. However, there was no history of fecal incontinence prior to the current febrile episode.

The patient was obese, 173 cm in height and weighing 148 kg, with a body mass index (BMI) of 49.5. He had been diagnosed with diabetes mellitus for over a decade, with a hemoglobin A1c level of 7.8%, which was drawn one month prior. His other medical history included an ankle ligament tear at the age of 20, urolithiasis at 22, and angina pectoris at 27. His regular medications consisted of metformin 1500 mg, glimepiride 2 mg, sitagliptin 50 mg, aspirin 200 mg, silodosin 4 mg, distigmine 5 mg, famotidine 20 mg, and magnesium oxide 1.5 g daily each. He had a three-decade history of cigarettes, smoking one pack per day, and denied consuming alcohol.

Upon arrival at the hospital, the patient was found in a debilitated state, so decontamination was initiated due to fecal and urinary contamination. The mental status remained clear. His vital signs at admission were as follows: temperature of 37.9℃, blood pressure of 106/56 mm Hg, heart rate of 92 bpm, respiratory rate of 22/minute, and oxygen saturation of 97% while breathing ambient air. Physical examination revealed no pallor or icteric conjunctiva. The pharynx appeared normal, and there were no signs of lymphadenopathy, thyroid swelling, or elevated jugular venous pressure. Due to obesity, it was hard to auscultate lung and heart sounds. His abdomen was non-tender and normoactive. There were no joint swellings or peripheral edema. An ulcer measuring 2 cm in diameter covered with eschar was observed on the outer lateral condyle of the left foot. While spontaneous movements were present in the upper extremities, the lower extremities demonstrated limited voluntary movement.

Fluid infusion was promptly initiated for his potential dehydration. The laboratory data are shown in Table 1.

**Table 1:** Laboratory data on admission WBC, white blood cells; Hb, hemoglobin; MCV, mean corpuscular volume; PLT, platelet; AST, aspartate aminotransferase; ALT, alanine aminotransferase; LDH, lactate dehydrogenase; ɤ-GTP, ɤ- glutamyl transpeptidase; Cre, creatinine; BUN, blood urea nitrogen; TP, total protein; Alb, albumin; CPK, creatine phosphokinase; CRP, C-reactive protein; HbA1c, hemoglobin A1c.

Lab item	Data	Normal range
WBC	13,080/µL	3,590 - 9,640
Hb	14.9 g/dL	13.2 - 17.2
MCV	89.2	85.6 - 102.5
PLT	19.8×10^4^/µL	14.8 - 33.9×10^4^
AST	173 IU/L	8 - 38
ALT	94 IU/L	4 - 44
LDH	393 IU/L	106 - 211
ɤ-GTP	23 IU/L	14 - 80
Cre	1.12 mg/dL	0.6 - 1.1
BUN	24.1 mg/dL	7.0 - 18.0
TP	6.5 g/dL	6.4 - 8.2
Alb	3.2 g/dL	3.4 - 5.0
CPK	4,603 IU/L	39 - 308
Na	137 mEq/L	136 - 145
K	3.2 mEq/L	3.5 - 5.1
Cl	102 mEq/L	98 - 107
Ca	8.4 mEq/L	8.5 - 10.1
Glucose	144 mg/dL	74 - 106
CRP	9.30 mg/dL	0 - 0.30
HbA1c	7.8%	4.5 - 6.2
D-dimer	3.7 µg/mL	0 - 1.0
Urine analysis		
pH	7.5	
Protein	±	
Glucose	-	
Urobilinogen	±	
Occult blood	+	
Leucocyte	3+	

**Figure 1:**
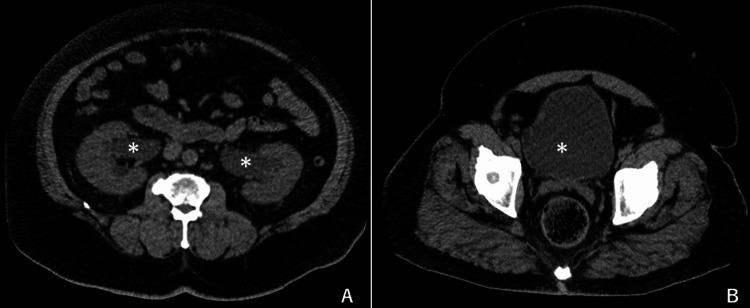
Abdominal CT on admission. (A) Bilateral hydronephrosis (asterisk). (B) Distended bladder (asterisk).

A head computed tomography (CT) scan yielded no abnormal findings. An abdominal CT revealed bilateral hydronephrosis, along with a distended bladder (Figure 1), which indicated any disorder of urination.

The prostate exhibited no signs of enlargement, measuring 41 x 51 mm. The electrocardiogram exhibited a complete right bundle branch block.

A preliminary diagnosis of urinary tract infection was made based on the existence of pyuria and the conspicuous absence of other signs suggesting alternative sources of infection. The patient was admitted, and cefmetazole 3 grams daily for suspected urinary tract infection was instituted. In addition, glycemic management with insulin was initiated.

On day two of admission, the patient's fever subsided, and his overall condition improved. All sets of blood cultures and a urine culture returned with negative results. However, on day four, the patient remained unable to wake up from the bed independently. He continued experiencing a lack of urinary urgency and was resorting to diaper defecation, although there was no fecal incontinence. The patient's residual urine volume was 167 ml. On day seven, the antibiotic treatment was successfully completed.

The results of manual muscle testing, deep tendon reflex assessments, and pathologic reflex evaluations are presented in Table 2.

**Table 2:** The results of manual muscle testing, deep tendon reflex assessments, and pathologic reflex evaluations ++: mild hyperreflexia; ±: decreased reflex.

Manual muscle test	
Sternocleidomastoid	5/5
Trapezius	4/4
Deltoid	4/4
Pectoralis major	4/4
Biceps brachii	5/5
Triceps brachii	5/5
Abductor pollicis brevis	5/5
Iliopsoas	4/4
Quadriceps femoris	4/4
Tibialis anterior	5/5
Triceps femoris	4/4
Deep tendon reflex	
Biceps brachii	++/++
Triceps brachii	++/++
Brachioradialis	++/++
Achilles tendon	±/±
Patellar tendon	++/±
Pathologic reflex	
Babinski	Flexion/flexion
Chaddock	-/-

Thermal hypoalgesia was seen in the right third finger to the radial side of the forearm, the left fifth finger to the ulnar side of the upper arm, and bilateral feet below the ankle joint, particularly intense in the dorsal surface of the halluces. There was no hypoalgesia in the chest or abdomen. Vibratory sensation in the lower extremities was reduced, and the tuning fork vibrations could be detected for five seconds. Regarding dyskinesia, both hands were able to perform grasp-release movements 10 times in 10 seconds. The prostate was not enlarged or tender. The anal sphincter reflex was intact, and thermal hypoalgesia was evident in the perianal area.

**Figure 2:**
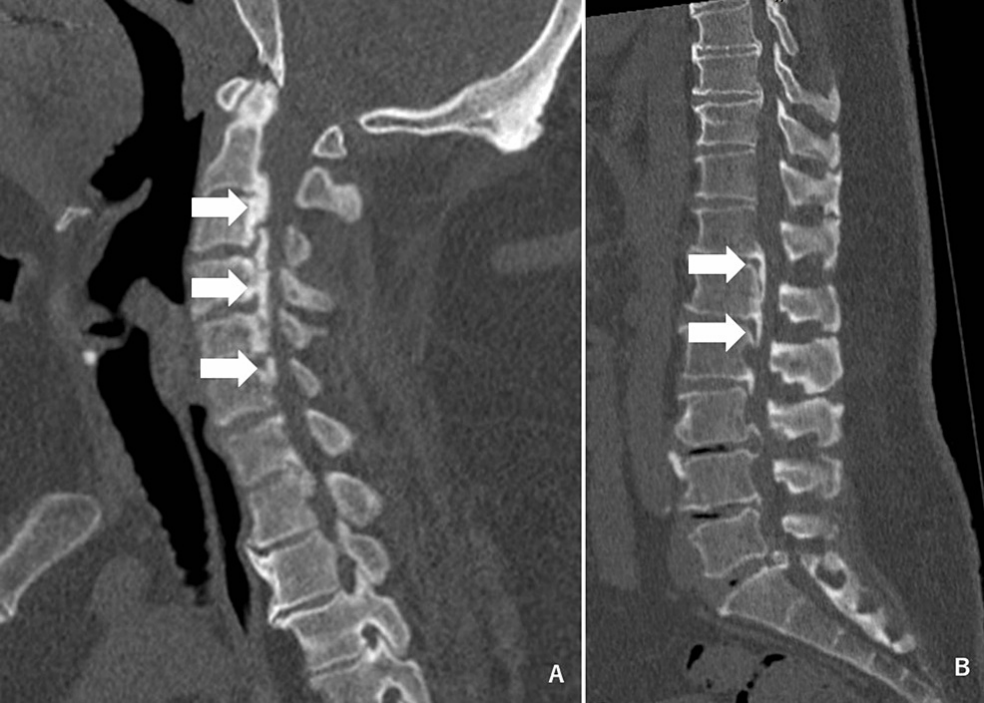
(A) Ossification of the posterior longitudinal ligament (OPLL) in the cervical spine (white arrow). (B) OPLL from the lower thoracic spine to the lumbar spine (white arrow).

An MRI imaging was attempted under the suspicion of a spinal disorder, but the test was not performed because of his large body size. A CT scan of the spine was performed, showing osteophytes on most vertebrae as well as multiple degenerated discs. Additionally, ossification of the posterior longitudinal ligament (OPLL) was seen in the cervical spine and from the lower thoracic spine to the lumbar spine (Figure 2A). The lumbar and sacral vertebrae had a narrowing of the spinal canal and neural foramen (Figure 2B).

We concluded that the patient had difficulty walking, because of mildly decreased muscle strength and obesity, and bladder and rectal disturbance due to neuropathy caused by diffuse idiopathic skeletal hyperostosis (DISH) and OPLL.

Spinal surgery was proposed but was eventually deferred pending an improvement in his obesity condition. Concurrently, we encouraged the patient to engage in rehabilitation and decrease his weight. Additionally, OPLL is designated by the Japanese government as a specific "rare and intractable disease," and we supported his application to alleviate his financial burden. Furthermore, preparations were made to assist the patient in obtaining long-term care insurance, thereby ensuring ongoing support for his daily life. Through dedicated rehabilitation efforts, the patient achieved the ability to transition to a wheelchair and walk 500 meters with the aid of a walker. On the 47th day, the patient was transferred to a specialized rehabilitation hospital. After a dedication to the rehabilitation hospital and a nursing care facility, he lost his weight to about 100 kg and undertook laminoplasty successfully.

## Discussion

The patient was confined to his bed at home due to multifactorial reasons. The patient's gait disturbance and urinary incontinence stemmed from myelopathy resulting from OPLL and DISH. In this context, urinary tract infections exacerbated his limited mobility, creating a scenario of acute-on-chronic mobility dysfunction. Truly, urinary tract infections, especially in frail patients, can independently lead to immobility acutely [1]. However, a thorough physical examination revealed chronic myelopathy in this case.

Many febrile patients complain of general malaise, which may result in immobility. However, some cases will find neuromuscular problems, which coexist with fever. For example, hypothyroidism, periodic tetraplegia, electrolyte abnormalities, demyelinating diseases such as Guillain-Barre syndrome, and central nervous system (CNS) disorders such as encephalitis, brain abscess, myelitis, and epidural abscesses may cause immobility in febrile patients. In other cases, physicians may be able to find the cause of patients' immobility in their social backgrounds: the patient might not have a cell phone, the patient might not be able to go outside due to stairs or other barriers, and the patient’s financial situation might prevent him from seeing a doctor.

If physicians make a tentative diagnosis of urinary tract infection in a patient with fever and pyuria without another apparent source of fever, continuing close monitoring is essential. Urinary tract infections in men are relatively uncommon than in women and should evoke some kind of suspicion. Male patients with urinary tract infections may harbor predisposing conditions, such as prostatitis, abnormal urinary tract structure, and urinary disorders [2]. Neglecting these conditions can result in treatment failure and recurrence. In this case, we employed both vertical tracing, to investigate the causes of the urinary tract infection, and horizontal tracing, to explore comorbidities such as diabetes [3]. This dual approach unveiled the patient's underlying conditions. The patient's medical history revealed a constellation of symptoms, including lower limb weakness, bilateral plantar numbness over several years, urinary incontinence for the past six months, reduced voiding, weak urinary output, and the sensation of residual urine. These clinical manifestations strongly point toward a condition with neurogenic bladder dysfunction. Therefore, a comprehensive neurological examination was warranted once the patient's condition stabilized.

This patient’s chronic walking difficulties over several years suggest underlying muscular or nerve dysfunction in the lower extremities. This patient's marked obesity and long-standing diabetes mellitus place him at a heightened risk for conditions such as lumbar spinal canal stenosis due to musculoskeletal change and diabetic neuropathy, both of which can contribute to dysuria. Additionally, the prolonged use of metformin places him at risk for vitamin B12 deficiency-induced lateral and posterior cord dysfunction. Finally, in this case, a constellation of symptoms, including muscle weakness, hypoalgesia, hyperreflexia in tendon reflexes, and hypoalgesia of the perineum, strongly indicates the spinal cord involvement rather than peripheral nerve origin. Immobility due to myelopathy and his diabetes may have precipitated a urinary tract infection. DISH and OPLL are linked to diabetes [4,5]. Delayed diagnosis of these conditions poses a threat to physical function. Physicians should be mindful that not all neuropathies in diabetic patients stem from diabetic peripheral polyneuropathy. The key to differential diagnosis lies in identifying muscle weakness, sensory disturbances along the dermadrome (not limited to the glove and stocking areas), hyperreflexia of the deep tendons, and bladder and rectal function disturbances.

Prior to surgical treatment for myelopathy, the patient needed long-term rehabilitation and nursing care. Physicians can help such patients by introducing appropriate social security and support systems. For example, OPLL is designated by the Japanese government as a specific “rare and intractable disease” [6]. The application of this support system aids with medical expenses. Additionally, long-term care insurance in Japan covers individuals aged 65 years or older, but patients with OPLL between the ages of 40 and 64 are also covered [7]. In this case, the patient could continue rehabilitation with long-term care insurance after the period covered by medical insurance had ended, enabling him to sustain his life until surgery. Being proficient in the operation of the system prevents the deterioration of patient’s quality of life and assists them in living better [8,9]. All these ultimately contribute to the quality of diagnosis and will likely be encompassed by the slogan of diagnostic excellence [10].

## Conclusions

In this case, a detailed analysis of history and physical examination revealed that the patient had complex problems apart from urinary tract infections. This patient was initially thought to be immobile due to obesity, urinary tract infection, and dehydration. However, in fact, it turned out that underlying myelopathy was discovered. Therefore, one message, in this case, is advocating the importance of organized diagnostic reasoning, the differential diagnosis based on it, and the dedicated medical history acquisition and physical examination skills, leading to the early detection of the true pathology. Another message is the importance of physicians being familiar with the social security system. Proficiency in system operation prevents the decline in patients' quality of life and facilitates their improved well-being. These factors collectively enhance the quality of diagnosis and are likely to align with the ethos of diagnostic excellence.
